# Blended online learning for oncologists to improve skills in shared decision making about palliative chemotherapy: a pre-posttest evaluation

**DOI:** 10.1007/s00520-023-07625-6

**Published:** 2023-02-23

**Authors:** D. W. Bos-van den Hoek, H. W. M. van Laarhoven, R. Ali, S. D. Bakker, A. Goosens, M. P. Hendriks, M. J. A. E. Pepels, D. Tange, F. Y. F. L. de Vos, A. J. van de Wouw, E. M. A. Smets, I. Henselmans

**Affiliations:** 1 Department of Medical Psychology, Amsterdam UMC, University of Amsterdam, Meibergdreef 9, PO Box 22660, 1100 DD Amsterdam, The Netherlands; 2 Quality of Care Program, Amsterdam Public Health Research Institute, Amsterdam, The Netherlands; 3 Cancer Center Amsterdam, Amsterdam, The Netherlands; 4 Department of Medical Oncology, Amsterdam UMC, University of Amsterdam, Meibergdreef 9, Amsterdam, The Netherlands; 5 Department of Medical Oncology, Zaans Medical Center, Zaandam, The Netherlands; 6 Department of Oncology, Red Cross Hospital, Beverwijk, The Netherlands; 7 Department of Medical Oncology, Northwest Clinics, Alkmaar, The Netherlands; 8 Department of Oncology, Elkerliek Hospital, Helmond, The Netherlands; 9 Dutch Federation of Cancer Patient Organizations (NFK), Utrecht, The Netherlands; 10 Department of Medical Oncology, University Medical Center Utrecht, Utrecht University, Utrecht, The Netherlands; 11 Department of Oncology, VieCuri Medical Center, Venlo, The Netherlands

**Keywords:** Shared decision making, Continuing education, Palliative care, Neoplasms, Oncologists, Communication

## Abstract

**Purpose:**

To improve shared decision making (SDM) with advanced cancer patients, communication skills training for oncologists is needed. The purpose was to examine the effects of a blended online learning (i.e. e-learning and online training session) for oncologists about SDM in palliative oncological care and to compare this blended format with a more extensive, fully in-person face-to-face training format.

**Methods:**

A one-group pre-posttest design was adopted. Before (T0) and after (T2) training, participants conducted simulated consultations (SPAs) and surveys; after the e-learning (T1), an additional survey was filled out. The primary outcome was observed SDM (OPTION12 and 4SDM). Secondary outcomes included observed SDM per stage, SPA duration and decision made as well as oncologists’ self-reported knowledge, clinical behavioural intentions, satisfaction with the communication and evaluation of the training. Additionally, outcomes of the blended learning were compared with those of the face-to-face training cohort. Analyses were conducted in SPSS by linear mixed models.

**Results:**

Oncologists (*n* = 17) showed significantly higher SDM scores after the blended online learning. The individual stages of SDM and the number of times the decision was postponed as well as oncologists’ beliefs about capabilities, knowledge and satisfaction increased after the blended learning. Consultation duration was unchanged. The training was evaluated as satisfactory. When compared with the face-to-face training, the blended learning effects were smaller.

**Conclusion:**

Blended online SDM training for oncologists was effective. However, the effects were smaller compared to face-to-face training. The availability of different training formats provides opportunities for tailoring training to the wishes and needs of learners.

## Introduction

For most patients with metastatic cancer, the primary goals of anti-cancer treatment are maintaining the quality of life and prolonging survival. However, treatment options have uncertain, possibly limited benefits with high burden. Alternatively, patients may choose foregoing anti-cancer treatment. Often, no single best treatment strategy exists. In this setting, shared decision-making (SDM) is required to provide care that matches patients’ values and preferences best. SDM involves four steps: (1) introducing choice, (2) explaining options with related pros and cons, (3) elucidating patients’ values and constructing preferences and (4) jointly making or postponing the decision [1]. SDM is advocated because of respect for patient autonomy [1, 2], reports of positive patient outcomes, including improved satisfaction and less decisional conflict [3], and patients’ wish to be involved in SDM [4].

Although physicians value SDM [5], observational studies show that SDM is not always visible in palliative cancer care [6–10]. Often, limited awareness is created about available treatment options and the option to refrain from chemotherapy [6, 7]. Patients do not always receive clear information about the survival benefit of palliative chemotherapy [8], nor are their values and appraisals of treatment option characteristics explicitly addressed [6, 9]. Lastly, patients’ preferred decision-making role is infrequently elicited, and the decision-making process is not matched accordingly [10].

Physician training is proposed to facilitate the implementation of SDM. Several communication skills training (CST) programs on SDM have been developed [11] and have been shown to improve SDM [12–14]. Blended learning formats, i.e. online learning with some level of learner control (e.g. over time, place or pace) combined with more traditional instructor-led synchronous learning [15], are increasingly adopted for CST because of their flexibility, richness and cost-effectiveness [16]. Online and blended CST, both with and without participant interaction, benefits cancer and palliative healthcare professionals [17], and its completion rate can be up to six times higher compared to traditional training [18]. Although a review comparing e-learning or blended learning with conventional learning suggests that e-learning may be at least as effective as conventional training, no definite conclusions can be drawn given the large heterogeneity across studies [19].

In response to the call for more research into the effects of different formats of CST about SDM [14, 20], a blended online learning format (4 hours) of a previously evaluated highly effective intensive, in-person face-to-face training (10 hours) on SDM in palliative oncological care [12, 13] was developed and evaluated. The aim of this study is to examine the effects of this blended online learning. We hypothesise that the blended online learning will improve observed SDM about palliative systemic treatment in simulated consultations. Secondary outcomes include observed SDM per stage, knowledge, clinical behavioural intentions, satisfaction with communication, consultation duration, decision made and evaluation of the blended learning. Additionally, we aimed to compare the effect of the blended online format with a more extensive in-person face-to-face training format, which was evaluated in a similar design.

## Materials and methods

The Human Ethics Committee at the Amsterdam UMC, location AMC, provided ethical clearance for the study, and local permission was obtained at all participating hospitals. The STROBE guidelines [21] were followed in this report.

### Design

**Fig. 1:**
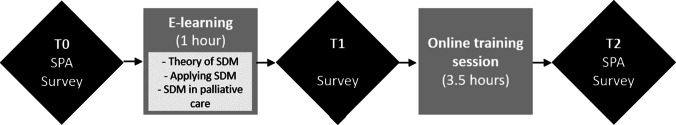
Study design

The study adopted a one-group pre-posttest design (Fig. 1). Participants engaged in standardised patient assessments (SPAs), i.e. simulated consultations with actors, at baseline (T0) and after the training (T2). In addition, participants filled out surveys at baseline (T0), after completing the e-learning (T1) and after the second SPA (T2).

### Setting and participants

Participants were medical oncologists (in training), who regularly have decision-making conversations with advanced cancer patients regarding starting, continuing or changing palliative systemic treatment.

### Sample size

Based on previously reported effect sizes [12, 13]), the study was powered to detect a large effect (Cohen’s *d* = 0.8). This required a sample size of fifteen oncologists (G*Power 3.1.9.2, *α* = 0.05, *β* = 0.80; paired *t*-test).

### Recruitment

Potential participants were contacted via medical oncology departments within hospitals, until at least fifteen oncologists were recruited. Interested oncologists were informed about the study by e-mail, received an information and informed consent letter, which was signed by all participants before the baseline SPA was performed. After attending the blended learning, oncologists received accreditation by the Netherlands Association of Internal Medicine.

### Training

The blended online learning consisted of two parts: an asynchronous component (e-learning) and a synchronous component with an instructor (online training session). We originally planned an in-person training session, but constrainedly switched to an online modality due to the COVID-19 restrictions. Both training parts addressed SDM knowledge, attitude (i.e. motivation and personal barriers) and skills (i.e. ability to apply the four stages of SDM). The e-learning consisted of three obligatory modules: (1) theory of SDM, (2) applying SDM and (3) SDM in palliative care, e.g. communication about prognosis and incorporating advance care planning, which were estimated to take 1 hour in total. The training session content was based on the previously evaluated face-to-face training [12, 13]. It adopted behaviour change techniques [22] among which providing instruction and prompting practice by role-play with professional actors according to the fishbowl working format, in which one learner practiced with one of the stages of SDM with an actor and the other participants observed and provided feedback [23]. The online training sessions were provided in small groups (*n* = 2–5) by an experienced trainer in a session of 3 hours. Afterwards, participants received a pocket-size card with the four SDM steps and example phrases as a follow-up prompt [22]. On average, the total training was estimated to take 4.5 hours. The blended learning was piloted in an in-person setting with six oncologists (in training) from three hospitals, after which small modifications were made.

### SPAs

Two different standardised patient assessment (SPA) cases, adopted from the previous trial [12], reflected a patient with either metastatic gastric or oesophageal cancer who met the oncologist to discuss the start of first-line palliative chemotherapy. For each participant, the cases were randomly assigned to either T0 or T2. Participants received a simulated medical file. Three experienced professional male actors (aged 57-64 years) played both roles. Two of the three actors also participated in the previous trial [12].The SPAs took place online due to COVID-19 restrictions and were video recorded (August 2020 to May 2021).

### Measurements

The outcomes were assessed at levels one (reaction, i.e. evaluation of training) and two (learning, i.e. self-reported changes or observed changes in simulated settings) of Kirkpatrick’s Model of Training Evaluation [24].

#### Sample characteristics

##### Participants

Oncologists reported their age, sex, whether or not they were in training, years of experience in medical oncology (including residency), number of palliative cancer patients in their care for the period of 1 month and receipt of communication skills training during medical school, residency and post education (yes/no). Besides these background characteristics, both oncologists’ perception of their patients’ attitude towards SDM and their own attitude were assessed with the Control Preference Scale (CPS, a 1-item measure with five different treatment decision-making roles [25]). The items were rearranged to reflect an active, shared or passive role of patients [26] or an informative, SDM or paternalistic role of oncologists [5].

##### SPAs

After each SPA, oncologists were asked how realistic and comparable to their clinical practice the simulated consultation was using four study-specific items with Likert scale responses (1–10).

#### Primary outcome

The primary outcome was the level of SDM as assessed from video-recorded SPAs using two instruments. First, the Observing Patient Involvement Scale (OPTION12), a widely used 12-item scoring instrument of physician communicative behaviour associated with SDM [27, 28]. Items are rated on a 5-point Likert scale (0: not observed–4: very high standard), and the sum score is transformed to reflect a total out of 100. Next to the general OPTION12 manual, a study-specific manual from the previous evaluation study was used [12]. Second, the 4SDM was used, an instrument developed by Henselmans et al. [12] based on the four-stage SDM model [1]. The 4SDM has eight items, which are coded on a 4-point Likert scale (0: not observed–3: observed and of high quality). Two blinded assessors rated the video-recorded consultations. The coding process consisted of training, calibration to achieve sufficient interrater reliability and independent coding. Since ICCs and kappa’s were not considered sufficient for independent coding, all SPAs were double coded and scores averaged or discussed until consensus was reached (Appendix 1).

#### Secondary outcomes

See Table 1 for a description of the secondary outcomes and how they were assessed.

**Table 1:** Secondary outcomes

Outcome	Time	Measures
Observed SDM per stage	T0, T2	Subscales of the 4SDM: • Setting the SDM agenda (2 items, range 0–6) • Informing about options (2 items, range 0–6) • Exploring values (2 items, range 0–6) • Making a decision (2 items, range 0–6)
Clinical behavioural intentions	T0, T1, T2	Subscales of the Continuing Professional Development (CPD; 12 items) reaction questionnaire [41]: • Intention to adopt a behaviour (2 items; range 1–7) • Social influence: perception of approval by persons significant to the individual (3 items; range 1–7) • Beliefs about capabilities: oncologists’ perceptions of facilitators and barriers (3 items; range 1–7) • Moral norm: feeling of personal obligation (2 items; range 1–7) • Beliefs about consequences: subjective probability that certain consequences will follow (2 items; range 1–7)
Knowledge about SDM	T0, T1	Self-developed knowledge test covering the content of the e-learning modules (12 items, range 0–12 right answers)
Satisfaction with SPA communication	T0, T2	Adjusted Patient Satisfaction Questionnaire (PSQ; 5 items; visual analogue scale (VAS); range: 0–100) [42] in a modified version for oncologists [43] and an additional sixth item on satisfaction with patient involvement in decision-making
SPA duration	T0, T2	Registered based on the video-recorded SPAs
Decision to start chemo	T0, T2	Registered based on observation of the SPAs and categorised into (a) start chemotherapy and (b) decision postponed
Evaluation of blended learning	T1, T2	Self-developed survey (19 items) on one or more of the separate elements of the blended learning: • Content (1: very bad–10: very good) • Usefulness (1: not useful at all–10: very useful) • Helpfulness to apply (even) more SDM (1: totally disagree–10: totally agree) • Perceived change in knowledge through e-modules (1: totally disagree–7: totally agree) • Time spent (0–15, 15–30, 30–45, 45–60 and over 60 min) • Recommending the training elements to colleagues (yes/no/maybe) • Expectation of colleagues to accept the elements (yes/no/maybe) • Perceived fit between/evaluation of combination of training elements (2 items; 1: very bad–10: very good) • Experiences with online instead of the in-person modality of the training session (4 items; range 1–10), transformed into: online modality is worse (1–4), equal (5–6) or better (7–10) than in-person modality • Preference of in-person over online modality (1: totally disagree–10: totally agree)

Abbreviations: *SDM*, shared decision-making; *SPA*, standardised patient assessment

### Comparison of training formats

For comparing different training formats, data (*n* = 31 oncologists) from a previously evaluated face-to-face training conducted in 2016 was used [12]. This training took 10 hours, including preparatory reading (1.5 hours), two small group training sessions with mainly role-play (3.5 hours each) and a booster session (1–1.5 hours, 6 weeks after the last training session). The face-to-face training was evaluated in a randomised controlled trial, in which both the intervention and the control group participated in SPAs and questionnaires. The eligibility criteria, SPAs, actors, coding instruments and questionnaire items (except from the items regarding clinical behavioural intentions and knowledge) were similar to those of the current study. Apart from the training format, there were additional differences between both trials: the previous trial (1) involved a different trainer, (2) involved different observers, (3) had SPA cases not randomly assigned to either T0 or T2, (4) had SPAs taking place in-person instead of online, (5) had a shorter average time between training and T2 (on average 11 days as opposed to 41 days) and (6) took place 5 years earlier. These differences warrant a cautious interpretation of the comparison.

### Statistical analyses

Linear mixed models (LMMs) were conducted in IBM SPSS Statistics 26 (IBM Corporation, Armonk, NY, USA) with time as an independent fixed effect. Separate analyses were conducted for the outcomes observed SDM (OPTION12 and 4SDM), the stages of SDM (4SDM), satisfaction with the conversation (PSQ), clinical behavioural intentions (CPD) and knowledge. For the dichotomous outcome decision made, a generalized estimating equation (GEE) model was used with time as an independent fixed effect. For each model, different repeated covariance types were compared, and the model with the lowest AIC was used. Cohen’s *d* was presented as a measure of effect size (*d* = 0.20 small, *d* = 0.50 medium, *d* = 0.80 large effects) [29]. The comparison between the two training formats was assessed in LMMs with time, condition and time*condition as fixed factors and, except from clinical behavioural intentions (CPD) and knowledge, the same outcomes as described above. First, the control group of the face-to-face training trial was used as the reference category, and second, the blended learning group was used as the reference category to compare the face-to-face training with the blended learning group.

## Results

After contacting 25 hospitals, seventeen oncologists from two academic and five non-academic hospitals participated in the evaluation. Of two respondents, the T0 SPA recording was missed due to technical issues, and one oncologist missed T1 after the e-learning (see Table 2 for participant and SPA characteristics).

**Table 2:** Participant and SPA characteristics

Participant characteristics (*n* = 17)		
Age in years, mean (SD)	42.82 (9.68)	
Gender,* n* (%) female	11 (64.7)	
Staff or resident, *n* (%) staff	12 (70.6)	
Type of hospital, *n* (%) academic	9 (52.9)	
Years of experience, mean (SD)	10.18 (9.53)	
Communication skills training during, *n* (%) yes		
Medical school	16 (94.1)	
Residency	12 (70.6)	
Post education^a^	5 (29.4)	
Role of patients in SDM, *n* (%)		
Active role	2 (11.8)	
Shared role	9 (52.9)	
Passive role	6 (35.3)	
Role of oncologist in SDM, *n* (%)		
Informative role	7 (41.2)	
Shared decision-making role	10 (58.8)	
Days between training and T2, mean (SD)	41.41 (23.43)	
SPA characteristics	T0 (*n* = 17)	T2 (*n* = 17)
Actor in SPA		
Actor A, *n* (%)	5 (29.4)	9 (52.9)
Actor B, *n* (%)	9 (52.9)	6 (35.3)
Actor C, *n* (%)	3 (17.6)	2 (11.8)
Case used in SPA, *n* (%), case 1	8 (47.1)	9 (52.9)
Perceived realism (1–10), mean (SD)		
Perceived realism	7.29 (1.72)	7.12 (2.29)
Perceived comparability	6.77 (1.68)	6.77 (1.68)
Influence of actor	4.06 (1.85)	4.76 (2.44)
Influence of online	5.88 (2.69)	5.47 (2.58)

^a^The five residents and one staff member indicated ‘not applicable’

Abbreviations: *SD*, standard deviation; *SDM*, shared decision-making; *SPA*, standardised patient assessment

### Effect of the blended online learning

The oncologists demonstrated significantly more SDM after the blended online learning as measured with both the OPTION12 (F(1, 26.436) = 17.181, *p* < 0.001) and the 4SDM (F(1, 28.818 = 20.544, *p* < 0.001) (Table 3). The effect size was large for both primary outcomes. In addition, SDM in all four stages (stage 1: F(1, 24.962) = 18.323, *p* < 0.001; stage 2: F(1, 15.000) = 24.380, *p* < 0.001; stage 3: F(1, 15.811) = 18.318, *p* = 0.001; stage 4: F(1, 16.130) = 5.283, *p* = 0.035), oncologists’ knowledge about SDM (F(1, 28.420) = 7.180, *p* = 0.012) and the satisfaction of oncologists with the conversation (F(1, 17.000) = 24.362, *p* < 0.001) improved after the blended online learning. Of the measures relating to clinical behavioural intentions, only oncologists’ *beliefs about capabilities* significantly improved after the blended learning (F(2, 37.668) = 5.593, *p* = 0.007); *intention* (F(2, 33.077) = 1.525, *p* = 0.233), *social influence* (F(2, 20.273) = 1.198, *p* = 0.322), *moral norm* (F(2, 33.493 = 1.517, *p* = 0.234) and *beliefs about consequences* (F(2, 31.150) = 0.398, *p* = 0.675) did not. The SPA duration did not change (F(1, 16.183) = 0.352, *p* = 0.561), and the decision was almost eight times more likely to be postponed after the blended learning (OR = 7.76, *p* = 0.039).

**Table 3:** Effect of blended learning; raw means and standard deviations at T0, T1 and T2 and parameter estimates and 95% CIs of the fixed effects in the mixed linear models on all outcomes

Outcome (range)	T0 (*n* = 15)^a^	T1 (*n* = 17)	T2 (*n* = 17)	*b* (95% CI)	Sig	*d*^b^
SDM OPTION12 (0–100)^c^	43.13 (12.29)	-	58.33 (8.57)	15.21 (7.67, 22.74)	0.000	1.01
SDM 4SDM (0–24)^c^	13.27 (4.31)	-	19.38 (3.47)	6.12 (3.36, 8.88)	0.000	1.10
Stage 1 Setting SDM agenda (0–6)	3.77 (1.18)	-	5.24 (0.75)	1.47 (0.76, 2.18)	0.000	1.04
Stage 2 Informing about options (0–6)	3.57 (1.74)	-	5.44 (0.83)	1.93 (1.10, 2.77)	0.000	1.20
Stage 3 Exploring values (0–6)	3.23 (1.19)	-	4.88 (1.17)	1.66 (0.84, 2.48)	0.001	1.04
Stage 4 Making a decision (0–6)	2.70 (2.02)	-	3.82 (1.81)	1.18 (0.09, 2.27)	0.035	0.56
Oncologist clinical behavioural intentions						
Intention (1–7)	6.24 (0.50)	6.28 (0.71)	-	0.03 (− 0.32, 0.37)	0.827	0.04
		-	6.50 (0.59)	0.27 (− 0.07, 0.60)	0.120	0.39
Social influence (1–7)	5.12 (1.08)	5.26 (0.67)	-	0.13 (− 0.37, 0.63)	0.602	0.13
		-	5.46 (0.53)	0.34 (− 0.21, 0.88)	0.213	0.31
Beliefs about capabilities (1–7)	5.63 (0.51)	5.73 (0.82)	-	0.00 (− 0.35, 0.36)	0.982	0.01
		-	6.04 (0.53)	0.41 (0.14, 0.69)	0.004	0.74
Moral norm (1–7)	6.09 (0.51)	6.25 (0.68)	-	0.17 (− 0.18, 0.52)	0.332	0.24
		-	6.38 (0.67)	0.29 (− 0.05, 0.64)	0.092	0.42
Beliefs about consequences (1–7)	6.21 (0.55)	6.19 (0.63)	-	0.02 (− 0.31, 0.35)	0.428	0.03
		-	6.27 (0.59)	0.15 (− 0.22, 0.52)	0.894	0.19
Oncologist knowledge (0–11)	8.41 (1.46)	9.50 (0.89)	-	1.09 (0.26, 1.92)	0.012	0.65
Oncologist satisfaction (0–100)	63.27 (8.98)	-	71.90 (7.34)	8.63 (4.94, 12.32)	0.000	1.20
SPA duration, mm:ss	30:43 (06:41)	-	29:59 (04:59)	− 0:55 (− 4:13, 2:22)	0.561	− 0.14
Decision postponed, *n* (%)^d^	10 (66.7)	-	16 (94.1)	2.05 (0.10, 3.99)	0.039	0.50

^a^Two recordings of SPAs were missing due to technical issues

^b^Cohen’s *d* was calculated by $b/(\sqrt{n}*\mathrm{SE}$)

^c^The correlation between the OPTION12 and the 4SDM was strong (T0: *r* = 0.92, *p* < 0.001; T2: *r* = 0.90, *p* < 0.001)

^d^Decision postponed was analysed by generalized estimating equations, *b* was ln(OR), and the *p*-value was based on the *Χ*^2^-statistic

Abbreviations: *4SDM*, four-step SDM instrument; *CI*, confidence interval; *OPTION12*, 12-item observing Patient Involvement Scale; *SDM*, shared decision-making; *SPA*, standardised patient assessment

### Evaluation of training

Except for three oncologists, all participants completed the three required e-learning modules. Oncologists assessed the e-learning with a 7.3 and the online training session with an 8.5 averagely (Table 4). About 60% would recommend the e-learning to colleagues, and about 90% would recommend the training session. Most participants indicated it took 15–30 min to complete an e-learning module, adding up to a total of 45–90 min for all three modules. When asked about the online modality of the training session, most respondents implied that its quality, usefulness and enjoyment were equal to an in-person modality and that it was more practical.

**Table 4:** Evaluation outcomes of training

	E-learning^a^							Online training session (*n* = 17)
	Theory of SDM (*n* = 16)	Applying SDM (*n* = 15)		SDM in palliative care (*n* = 16)		Overall (*n* = 16)		
Rating, mean (SD)								
Content (1–10)	7.38 (0.81)	7.53 (0.83)		7.13 (0.89)		7.31 (0.79)		8.47 (0.80)
Usefulness (1–10)	7.50 (0.89)	7.40 (0.99)		7.25 (0.93)		7.38 (0.81)		8.35 (0.86)
Helped applying SDM, mean (SD); agreement (1–10)^b^	7.95 (2.08)	8.29 (1.23)		7.95 (1.47)		-		8.24 (0.97)
Knowledge gain, mean (SD); agreement (1–7)	5.56 (0.72)	5.80 (0.68)		5.25 (0.93)		-		-
Time spent (in minutes), *n* (%)								
0–15	7 (43.8)	4 (26.7)		5 (31.3)		-		-
15–30	8 (50.0)	7 (46.7)		10 (62.5)		-		-
30–45	1 (6.3)	3 (20.0)		-		-		-
45–60	-	1 (6.7)		1 (6.3)		-		-
Recommendation to colleagues, *n* (%)								
Yes	-	-		-		10 (62.5)		15 (88.2)
Maybe	-	-		-		5 (31.3)		2 (11.8)
No	-	-		-		1 (31.3)		-
Would colleagues use training to improve knowledge (e-learning)/skills (training session), *n* (%)								
Yes	-	-		-		8 (50.0)		15 (88.2)
Maybe	-	-		-		6 (37.5)		2 (11.8)
No	-	-		-		2 (12.5)		-
Assessment of combination e-learning and online training session, mean (SD)								
Fit (1–10)	7.41 (1.12)							
Quality combination (1–10)	7.59 (1.18)							
Assessment of online instead of in-person modality of training session								
	Median (IQR)		Worse, *n* (%)		Equal, *n* (%)		Better, *n* (%)	
Quality (1–10)	6 (5.0–8.0)		2 (11.8)		9 (52.9)		6 (35.3)	
Usefulness (1–10)	6 (5.0–7.0)		-		11 (64.7)		6 (35.3)	
Enjoyment (1–10)	6 (4.5–7.0)		4 (23.5)		8 (47.1)		5 (29.4)	
Practicality (1–10)	8 (7.0–9.0)		-		3 (17.6)		14 (82.4)	
Preferring in-person format (1–10)	5 (4.5–7.0)		-		-		-	

^a^One respondent was missing in the e-learning assessment

^b^In the e-learning evaluation, this item was assessed on a scale of 1–7 and transformed to reflect a range of 1–10

Abbreviations: *IQR*, interquartile range; *SDM*, shared decision-making; *SD*, standard deviation

### Comparison between different training formats

Table 5 presents the raw means of the current blended online training group (*n* = 17) as well as of the face-to-face training group (*n* = 15) and the control group (*n* = 16) of the previous trial [12]. Except for stage 3 of SDM (F(2, 84.537) = 2.232, p = 0.114) and satisfaction (F(2, 48.000) = 2.430, *p* = 0.099), the interaction between time and condition (previous control group, previous face-to-face training group and current blended learning group) was significant for all outcomes, among which the primary outcomes (OPTION12: F(2, 88.210) = 6.396, *p* = 0.003; 4SDM: F(2, 84.132) = 7.681, *p* = 0.001) and the three other stages of SDM (stage 1: F(2, 90.014) = 5.829, *p* = 0.004; stage 2: F(2, 73.276) = 6.203, *p* = 0.003; stage 4: F(2, 46.313) = 5.301, *p* = 0.008). Post hoc comparisons showed that the group which received the blended learning did not differ significantly from the control group of the previous study on any of the outcomes (Table 6). The differences between the blended learning group and the previous control group on the primary outcomes were of small to medium size, while the differences between the face-to-face training and the control group were large. When comparing the two formats with each other, the blended learning format showed a significantly smaller effect compared to the face-to-face format on the primary outcomes. Except for stage 4, the two formats did not differ significantly on the other individual SDM stages nor on oncologist satisfaction with the conversation.

**Table 5:** Comparison of training formats; raw means and standard deviations pre and post intervention on all outcomes

	Blended learning 2020/2021 (*n* = 17)		Reference group 2015/2016 (*n* = 16)		Face-to-face training 2015/2016 (*n* = 15)	
Outcome (range)	Pre	Post	Pre	Post	Pre	Post
SDM OPTION12 (0–100)	43.13 (12.28)	58.33 (8.57)	34.63 (11.56)	44.14 (11.46)	35.63 (12.36)	63.96 (8.77)
SDM 4SDM (0–24)	13.27 (4.31)	19.38 (3.47)	11.75 (5.13)	14.56 (3.94)	11.70 (3.86)	22.07 (2.03)
Stage 1 Setting SDM agenda (0–6)	3.76 (1.18)	5.24 (0.75)	2.94 (1.44)	3.44 (1.47)	2.90 (1.00)	5.30 (0.68)
Stage 2 Informing about options (0–6)	3.57 (1.74)	5.44 (0.83)	2.81 (1.33)	3.78 (1.05)	2.83 (1.19)	5.83 (0.52)
Stage 3 Exploring values (0–6)	3.23 (1.19)	4.88 (1.17)	2.81 (1.80)	3.81 (1.22)	2.93 (1.75)	5.40 (1.17)
Stage 4 Making a decision (0–6)	2.70 (2.02)	3.82 (1.81)	3.19 (1.76)	3.53 (1.23)	3.03 (1.39)	5.53 (0.86)
Oncologist satisfaction (0–100)	63.27 (8.98)	71.90 (7.34)	58.68 (14.71)	64.95 (9.93)	55.02 (12.96)	68.56 (8.65)

Abbreviations: *4SDM*, four-step SDM instrument; *SDM*, shared decision-making; *OPTION12*, 12-item observing Patient Involvement Scale

**Table 6:** Post hoc comparisons of training formats; parameter estimates and 95% CIs of the fixed effects in the mixed linear models on all outcomes

	Face-to-face training vs control			Blended learning vs control			Face-to-face vs blended format		
Outcome (range)	*b* (95% CI)	Sig	*d*^a^	*b* (95% CI)	Sig	*d*^a^	*b* (95% CI)	Sig	*d*^a^
SDM OPTION12 (0–100)	18.83 (8.14, 29.52)	0.001	1.18	5.70 (− 4.86, 16.27)	0.286	0.41	13.13 (2.39, 23.86)	0.017	0.85
SDM 4SDM (0–24)	7.55 (3.72, 11.39)	0.000	1.33	3.30 (− 0.49, 7.10)	0.087	0.65	4.25 (0.39, 8.11)	0.031	0.78
Stage 1 Setting SDM agenda (0–6)	1.90 (0.79, 3.01)	0.001	1.25	0.97 (− 0.12, 2.06)	0.081	0.65	0.93 (− 0.18, 2.04)	0.099	0.69
Stage 2 Informing about options (0–6)	2.03 (0.88, 3.18)	0.000	1.26	0.91 (− 0.24, 2.05)	0.118	0.57	1.13 (− 0.04, 2.29)	0.057	0.67
Stage 3 Exploring values (0–6)	1.47 (0.09, 2.85)	0.038	0.81	0.65 (− 0.72, 2.02)	0.348	0.42	0.82 (− 0.51, 2.15)	0.223	0.49
Stage 4 Making a decision (0–6)	2.16 (0.81, 3.50)	0.002	1.30	0.83 (− 0.51, 2.16)	0.218	0.48	1.32 (0.09, 2.54)	0.036	0.70
Oncologist satisfaction (0–100)	7.26 (0.52, 14.01)	0.035	0.57	2.36 (− 4.18, 8.89)	0.472	0.21	4.91 (− 1.00, 10.81)	0.100	0.43

^a^Cohen’s *d* was calculated by$b/{\mathrm{SD}}_{\mathrm{pooled}}$

Abbreviations: *4SDM*, four-step SDM instrument; *CI*, confidence interval; *SDM*, shared decision-making; *OPTION12*, 12-item observing Patient Involvement Scale

## Discussion

By means of a one-group pre-posttest design, we showed a large and significant effect of the blended learning on observed SDM in standardised patient assessments. To the best of our knowledge, this is the first positively evaluated blended online learning for oncologists about SDM. In addition, the blended learning improved oncologists’ skills in all four SDM stages, their knowledge about SDM, beliefs about capabilities, satisfaction with the consultation and increased the frequency of postponing the decision. The blended learning did not increase the consultation duration. Oncologists evaluated the blended online learning as satisfactory and did not clearly express a preference for either an online or a face-to-face modality. Secondly, we compared CST formats by contrasting the 4-hour blended online training to a previously evaluated 10-hour face-to-face training. Taking limitations into account when comparing the two training formats, the effect of the blended learning on SDM appears to be smaller compared to the face-to-face training.

As stated in the Introduction section, several SDM training programs for oncologists (in training) and internal medicine residents have large training effects. This study shows that SDM skills can also improve with training in a blended online format, partly without an instructor. Although the low response rate might suggest little enthusiasm for the blended learning, participating oncologists graded the blended online learning with an average of 7.9 (range 1–10). Probably, the low response rate was due to the emergency situation during the first months of the COVID-19 pandemic during which the study was performed. The online modality was well appreciated, especially from a practical perspective. All this is promising from an efficiency and implementation point of view, especially taking into account the physical restrictions during the COVID-19 pandemic era [30].

The results tentatively suggest that the more intensive 10-hour face-to-face training format is more effective than the 4-hour blended online learning format. Previous research regarding training duration yielded mixed results: while some research shows that longer CST, for example at least 1 [31] or 3 [32] days, is most successful, other research demonstrates that training less than 10 hours is as successful as longer training [33]. Besides, a review concluded that blended learning formats may be more effective than traditional learning [19]. Strong evidence for effective features of CST regarding format, intensity and content is not yet available [20]. Nevertheless, as both the face-to-face and the blended learning format evaluations showed large effects on SDM skills, albeit in different study designs, the results call for a personalised training approach, using the right ingredients in different situations and for different learners.

A first issue in the comparison of training formats may be the changing SDM zeitgeist. The OPTION12 scores were significantly higher at baseline in the blended learning evaluation (2020/2021) as compared to the face-to-face training evaluation (2015/2016). This might imply that, over time, SDM has become better incorporated in clinical practice due to physicians better applying SDM or patients being more aware of SDM principles. Secondly, the duration between the last training moment and the follow-up SPA was significantly longer in the blended format. When adjusting for this duration, the differences between the two formats decreased. This may indicate that the training effects decrease over time, probably hindering the transfer of skills in clinical practice. Furthermore, it has yet to be established what the effects are of online SPAs, as were conducted in the current study, rather than in-person SPAs, as were conducted in the previous study, on observed SDM skills. Possibly, participants can demonstrate the learnt skills better in live SPAs than in online SPAs. In line, a study on Objective Structural Clinical Examination found that those participating in online examinations performed worse than those participating onsite [34].

Despite all inherent limitations, the comparison of training formats may be regarded as a strength of the current study. Such comparisons are rare in literature and contribute to the better use of research data. Another strength is the evaluation of training outcomes on different levels of Kirkpatrick’s model, i.e. the level of reaction and learning. On the level of learning, we both evaluated if the participant ‘knows how’ (e.g. the knowledge test) and ‘shows’ (the SPA) in terms of Miller’s model of clinical competence [35]. The design of the current study has limitations as well. Different training intensities (10 versus 4 hours) and formats (face-to-face versus e-learning and online training session) were simultaneously compared, which hinders understanding about which effective ingredient has which effect. Secondly, the blended online learning was not evaluated in a randomised controlled trial, and given the lack of randomization and absence of a true parallel control group, confounding explanations for its effect cannot be excluded. Also, participants may have learned unintentionally from the baseline SPA. Indeed, in the previous face-to-face training evaluation, the control group also significantly improved their SDM skills. Thirdly, the study population may not be completely representative, as possibly only highly motivated oncologists participated in this COVID-19 era. Lastly, the trial was powered to establish large training effects, which were demonstrated in this study design. However, when comparing the blended learning with the control group of the previous trial, small to medium effect sizes were found, for which the trial was not powered. Nevertheless, these effects may imply a clinically relevant change in SDM behaviour.

Next, research steps should be to conduct non-inferiority trials in robust study designs, comparing different intensities and formats of SDM training to find the ideal dose–response balance. Ideally, research also establishes the effects of training on behaviour of oncologists in the clinical setting and on patient outcomes [14, 20], including both observer and patient-reported outcomes. Since the patient may experience more involvement than observers recognise [36], different methods, e.g. conversation analysis [37], or different instruments, e.g. MAPPIN’SDM that includes observers’ as well as physicians’ and patients’ perspective [38], could be deployed to perceive insight in patient experiences. Future research should also demonstrate if the acquired skills are retained over time and whether differences between training formats continue to exist. Additionally, SDM increasingly takes place in multiple conversations with multiple healthcare professionals, also referred to as interprofessional SDM [39]. This is supported by this study’s finding that, after training, significantly more decisions were postponed, suggesting that patients would need another conversation about the treatment decision, either with the oncologist or another healthcare professional. It was previously stated that for optimal implementation of SDM in practice, the interprofessional nature of SDM should be acknowledged [40]. Given that current as well as previous research has shown that SDM skills of oncologists can be improved through training, other healthcare professionals in the SDM process may benefit from such training.

In conclusion, the blended online SDM training for oncologists was found to be effective. This is promising given the flexible, rich and cost-effective nature of blended learnings, especially in pandemic times. These findings are not entirely conclusive, since a pre-posttest evaluation design was adopted and the comparison with data from a previous study involving a face-to-face training showed smaller effect sizes for the blended online training. Nevertheless, opportunities arise for tailoring training formats to the wishes and needs of learners.

## Data Availability

Data, material and/or codes are available upon reasonable request, except for the video-recorded SPAs (i.henselmans@amsterdamumc.nl).

## Appendix 1

### Assessor training

Two psychologists with experience in using the OPTION12 and 4SDM [13] and providing communication skills training in a medical setting restudied the manuals and discussed them with two researchers (IH, DB). They independently rated three video-recorded SPAs from the previous evaluation study with SPAs [12]. After rating these video recordings, the assessors compared their scores and discussed inconsistencies to reach a common understanding of the items and response categories. One of the researchers (DB) facilitated these discussions.

### Assessor calibration

The assessors repeatedly double coded sets of five SPAs with both the OPTION12 and 4SDM. Interrater reliability (IRR) was calculated after each set. The IRR of the OPTION12 and 4SDM was considered sufficient if the intraclass correlation (ICC) and the average weighted kappa (*κ*) across items were higher than 0.60 for each item (reflecting substantial agreement) [44]. Κappas were prevalence-adjusted by balancing the matrix [45] if needed when row and column totals contained zeroes due to the low number of coded consultations and skewed distributions of ratings within items. When IRR was insufficient, scores of items with low *κ* were discussed and the study-specific manuals extended if needed. After the first set of SPAs (*n* = 5), the IRR was considered moderate for the OPTION12 (ICC = 0.76, *κ* = 0.58) and substantial for the 4SDM (ICC = 0.94, *κ* = 0.63). After coding the second set of SPAs (*n* = 5), the IRR was considered moderate to sufficient (OPTION12: ICC = 0.65, *κ* = 0.49; 4SDM: ICC = 0.86, *κ* = 0.50). The third set showed no improvement: the IRR was still moderate to sufficient (OPTION12: ICC = 0.87, *κ* = 0.55; 4SDM: ICC = 0.71, *κ* = 0.50) (see Appendix Table 7 for more details).

### Double coding SPAs

As the ICCs and kappas were not considered sufficient for independent coding after three calibration rounds, the remaining (*n* = 17) SPAs were coded double. After each sixth consultation, the items with scores > 1 point difference were discussed until consensus was reached and study-specific manuals extended if required. The scores with 1 point difference between the assessors were averaged.

### Overall IRR

The overall ICC between the assessors of the 32 SPAs was 0.868 (OPTION12) and 0.915 (4SDM). The overall average kappas of the OPTION12 and the 4SDM were both 0.62, reflecting substantial agreement. Of the OPTION12, five items had *κ* < 0.60 and of the 4SDM, two items. The observed percentage agreement was 64.6% for the OPTION12 and 66.0% for the 4SDM. One assessor seemed more strict than the other on scoring the OPTION12 (e.g. T0: *μ*_1_ = 41.39, SD_1_ = 13.20; *μ*_2_ = 44.44, SD_2_ = 12.62). However, paired sample *t*-tests between both assessors showed no significant differences (two-sided *p*-values) between the total scores of the OPTION12 (T0: *p* = 0.153; T2: *p* = 0.089) and 4SDM (T0: *p* = 0.935; T2: *p* = 0.079), indicating no assessor bias.

**Table 7:** Interrater reliability (IRR) in the calibration phase

	Set 1 (*n* = 5)			Set 2 (*n* = 5)			Set 3 (*n* = 5)			Overall (*n* = 15)		
	% agree	*κ*	ICC	% agree	*κ*	ICC	% agree	*κ*	ICC	% agree	*κ*	ICC
OPTION12	56.7	0.58	0.76	56.7	0.49	0.65	60.0	0.55	0.87	57.8	0.54	0.71
Item 1	40.0	0.44		60.0	0.48		40.0	0.33		46.7	0.46	
Item 2	60.0	0.69		80.0	0.62		40.0	0.55		60.0	0.64	
Item 3	80.0	0.84^a^		60.0	0.33^a^		100.0	1.00^a^		80.0	0.79^a^	
Item 4	80.0	0.74		60.0	0.67		60.0	0.64		66.7	0.70	
Item 5	60.0	0.74		40.0	0.40		80.0	0.74		60.0	0.66	
Item 6	40.0	0.23		20.0	0.00		80.0	0.58		45.7	0.27	
Item 7	20.0	0.38		40.0	0.33		20.0	0.14		26.7	0.27	
Item 8	80.0	0.78		40.0	0.48		40.0	0.14		53.3	0.30	
Item 9	60.0	0.44		80.0	0.55		80.0	0.74		73.3	0.43	
Item 10	100.0	1.00^a^		100.0	1.00^a^		100.0	1.00^a^		100.0	1.00^a^	
Item 11	20.0	0.00		60.0	0.75		40.0	0.47		40.0	0.48	
Item 12	40.0	0.63		40.0	0.21		40.0	0.29		40.0	0.47	
4SDM	67.5	0.63	0.94	55.0	0.50	0.86	62.5	0.50	0.71	61.7	0.55	0.87
Item 1	60.0	0.38		80.0	0.69^a^		80.0	0.74		73.3	0.53	
Item 2	80.0	0.76		60.0	0.55^a^		40.0	0.21		60.0	0.44	
Item 3	100.0	1.00		60.0	0.44		80.0	0.74		80.0	0.78	
Item 4	100.0	1.00		40.0	0.40		100.0	1.00		80.0	0.79	
Item 5	60.0	0.58		20.0	0.00		40.0	0.12^a^		40.0	0.35	
Item 6	100.0	1.00		40.0	0.48		60.0	0.55		66.7	0.68	
Item 7	40.0	0.35		60.0	0.57		20.0	0.00		40.0	0.42	
Item 8	0.0	0.00		80.0	0.84		80.0	0.69		53.3	0.42	

^a^Prevalence-adjusted kappa (PAK)

Abbreviations: *4SDM*, four-step SDM instrument; *ICC*, intraclass correlation; *OPTION12*, 12-item observing Patient Involvement Scale
